# Correction: Adverse clinical outcomes in people at clinical high-risk for psychosis related to altered interactions between hippocampal activity and glutamatergic function

**DOI:** 10.1038/s41398-021-01731-x

**Published:** 2021-11-30

**Authors:** Paul Allen, Emily J. Hird, Natasza Orlov, Gemma Modinos, Matthijs Bossong, Mathilde Antoniades, Carly Sampson, Matilda Azis, Oliver Howes, James Stone, Jesus Perez, Matthew Broome, Anthony A. Grace, Philip McGuire

**Affiliations:** 1 Department of Psychology, University of Roehampton, London, UK; 2 Department of Psychosis Studies, Institute of Psychiatry, Psychology and Neuroscience, King’s College London, London, UK; 3 Icahn School of Medicine, Mount Sinai Hospital, New York, NY USA; 4 National Institute of Health Research Biomedical Research Centre at South London and Maudsley National Health Service Foundation Trust, London, UK; 5 Liu Lab, Harvard Medical School, Athinoula Martinos Center for Biomedical Imaging, Massachusetts General Hospital, Boston, MA USA; 6 Department of Radiology, Xuanwu Hospital, Capital Medical University, Beijing, China; 7 Lab for Precision Brain Imaging, Department of Neuroscience, Precision Brain Imaging Lab, Medical University of South Carolina, Charleston, SC USA; 8 Department of Neuroimaging, Institute of Psychiatry, King’s College London, London, UK; 9 MRC Centre for Neurodevelopmental Disorders, King’s College London, London, UK; 10 Department of Psychiatry, Brain Center Rudolf Magnus, University Medical Center Utrecht, Utrecht, The Netherlands; 11 Medical Research Council London Institute of Medical Sciences, Hammersmith Hospital, London, UK; 12 Institute of Clinical Sciences, Faculty of Medicine, Imperial College London, London, UK; 13 CAMEO Early Intervention in Psychosis Service, Cambridgeshire and Peterborough NHS Foundation Trust, Cambridge, UK; 14 School of Psychology, University of Birmingham, Birmingham, UK; 15 Departments of Neuroscience, Psychiatry and Psychology, University of Pittsburgh, Pittsburgh, PA USA

**Keywords:** Predictive markers, Schizophrenia, Molecular neuroscience

Correction to: *Translational Psychiatry* 10.1038/s41398-021-01705-z, published online 10 November 2021

The original version of this article unfortunately contained a spelling mistake in the name of Anthony A. Grace. The authors apologize for the error. The original article has been corrected.

