# Supplementary material for: Adverse clinical outcomes in people at clinical high-risk for psychosis related to altered interactions between hippocampal activity and glutamatergic function
Source: Transl Psychiatry. 2021 Nov 10;11:579. doi: 10.1038/s41398-021-01705-z (PMC8580992; doi:10.1038/s41398-021-01705-z)
Supplement: Supplementary file 1 — Supplemental Material [file 41398_2021_1705_MOESM1_ESM.docx]

**Supplementary material**

Supplementary figure 1 summarises the ^1^H-MRS and fMRI measures. Supplementary table 1 summarises an assessment of the difference in baseline symptom severity between CHR subjects who were analysed in the functional analysis, and those who were not assessed for functioning as they were too unwell. Supplementary tables 2, 3 and 4 summarise the MRS quality check analysis. We tested the Group x ^1^H-MRS Glutamate concentration interaction on hippocampal activity (Supplementary table 5). We also tested the interaction between Group and ^1^H-MRS Glx interactions on activity during neutral > standard oddball trials (Supplementary table 6).


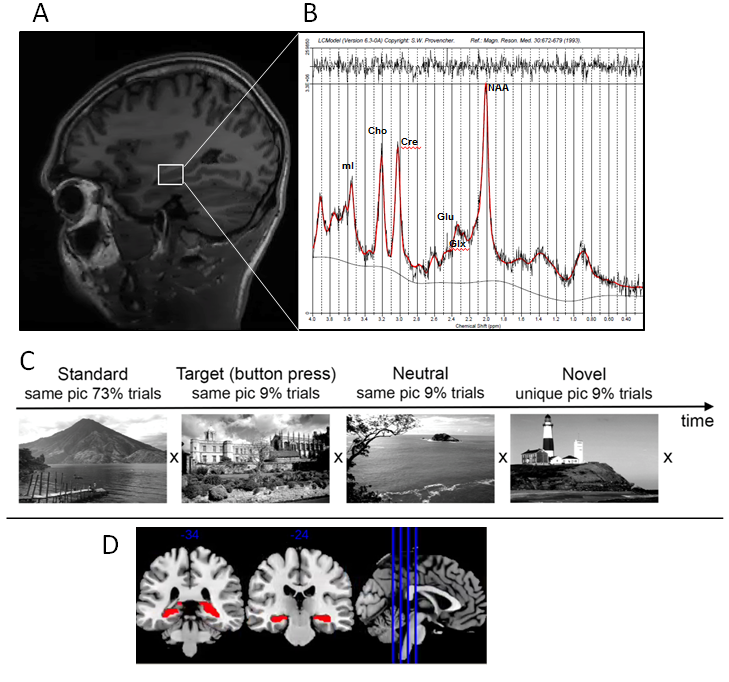


**Supplementary figure 1: ^1^H-MRS and fMRI measures**. An example of ^1^H-MRS voxel placement in the left hippocampus (A) and the ^1^H-MRS spectrum obtained from this voxel (black line) and the overlay of the spectral fit (red line) (B). mi indicates *myo-*inositol; Cho, choline; Cre, creatine; Glu, glutamate; Glx, combined measure of glutamine and glutamate; NAA, *N-*acetylasparate; ppm, parts per million. (C): the fMRI task paradigm and (D) Region-of-interest mask of the bilateral hippocampus used for small volume correction on the fMRI x MRS analysis.

**Supplementary table 1:** Results of independent samples t-test assessing baseline clinical scores in CHR subjects who were assessed at follow-up compared to CHR subjects who were too unwell to be assessed at follow-up:

| **Clinical measure** | **Group** | | |
| --- | --- | --- | --- |
|  | **CHR subjects assessed at follow-up**  **(N=52)** | **CHR subjects too unwell to be assessed at follow-up**  **(N=3)** | ***P*** |
| Positive CAARMS, mean (SD) | 10.35  (4.28) | 6.3  (2.51) | 0.09 |
| Negative CAARMS, mean (SD) | 5.88  (4.16) | 3  (1.41) | 0.14 |
| Total CAARMS, mean (SD) | 44.16  (20.05) | 35  (33.94) | 0.77 |

**Supplementary table 2:** Scan Quality Parameters and Voxel Tissue Composition: HC vs CHR Subjects.

| **Measure** | **HC (N=31)** | | **CHR (N=75)** | | **HC vs. CHR** | |
| --- | --- | --- | --- | --- | --- | --- |
|  | **Mean** | **SD** | **Mean** | **SD** | **t** | **P** |
| SNR | 13.94 | 2.76 | 12.93 | 2.84 | 1.69 | .09 |
| FWHM | .07 | .02 | 0.07 | 0.01 | -.74 | .47 |
| Line width | 8.35 | 2.22 | 8.68 | 1.8 | -.74 | .47 |
| Voxel GM | 0.64 | 0.07 | 0.64 | 0.06 | -.2 | .84 |
| Voxel WM | 0.33 | 0.08 | 0.33 | 0.07 | .44 | .66 |
| Voxel CSF | 0.03 | 0.02 | 0.04 | 0.02 | -1.09 | .28 |
| Glutamate % CRLB | 9.13 | 1.31 | 9.51 | 1.95 | -1.18 | .24 |
| GLx % CRLB | 10.1 | 2.55 | 10.23 | 2.57 | -.24 | .81 |
| Myo-inositol % CRLB | 6.03 | 1.54 | 5.89 | 1.58 | .42 | .67 |
| NAA % CRLB | 3.84 | 0.93 | 3.97 | 1.31 | -.59 | .55 |
| Total choline % CRLB | 3.97 | 0.89 | 4.32 | 2.22 | -1.18 | .24 |
| Creatine % CRLB | 3.84 | 0.69 | 4.01 | 0.89 | -1.09 | .28 |

HC, Healthy Control; CHR, Clinical High-Risk; SNR, signal to noise ratio; FWHM, full-width-half-maximum; GM, grey matter; WM: white matter; CSF, cerebrospinal fluid; CRLB, Cramer-Rao Lower Bounds; Glx, combined measure of glutamine and glutamate; NAA, N-acetylaspartate.

**Supplementary table 3:** Scan Quality Parameters and Voxel Tissue Composition: CHR-NT vs CHR CHR-T subjects.

| **Measure** | **CHR-NT (N=63)** | | **CHR-TR (N=12)** | | **CHR-NT vs. CHR-T** | | | |
| --- | --- | --- | --- | --- | --- | --- | --- | --- |
|  | **Mean** | **SD** | **Mean** | **SD** | | **t** | **P** |  |
| SNR | 12.98 | 2.98 | 12.67 | 1.67 | | .47 | .65 |  |
| FWHM | .07 | .01 | .06 | .01 | | 1.35 | .18 |  |
| Line width | 8.8 | 1.85 | 8.04 | 1.41 | | 1.35 | .18 |  |
| Voxel GM | .64 | .06 | .63 | .07 | | .29 | .78 |  |
| Voxel WM | .32 | .07 | .33 | .07 | | -.35 | .73 |  |
| Voxel CSF | .04 | .02 | .03 | .02 | | .23 | .82 |  |
| Glutamate % CRLB | 9.55 | 1.99 | 9.33 | 1.72 | | .39 | .71 |  |
| GLx % CRLB | 10.19 | 2.59 | 10.42 | 2.58 | | -.28 | .78 |  |
| Myo-inositol % CRLB | 5.92 | 1.51 | 5.75 | 1.96 | | .28 | .78 |  |
| NAA % CRLB | 3.92 | 1.39 | 4.25 | 0.62 | | -1.31 | .19 |  |
| Total choline % CRLB | 4.4 | 2.39 | 3.92 | 0.79 | | 1.28 | .21 |  |
| Creatine % CRLB | 4.03 | 0.93 | 3.92 | 0.67 | | .51 | .62 |  |

CHR-NT, Clinical High-Risk No Transition; CHR-T, Clinical High-Risk Transition; signal to noise ratio; FWHM, full-width-half-maximum; GM, grey matte WM: white matter; CSF, cerebrospinal fluid; CRLB, Cramer-Rao Lower Bounds; Glx, combined measure of glutamine and glutamate; NAA, N-acetylaspartate.

**Supplementary table 4:** Scan Quality Parameters and Voxel Tissue Composition: CHR-good vs CHR-poor.

| **Measure** | **CHR-good (N=17)** | | **CHR-poor (N=35)** | | **CHR-good vs. CHR-poor** | |
| --- | --- | --- | --- | --- | --- | --- |
|  | **Mean** | **SD** | **Mean** | **SD** | **t** | **P** |
| SNR | 11.59 | 2.92 | 12.97 | 2.39 | -1.69 | .1 |
| Line width | 8.96 | 2.27 | 8.83 | 1.78 | .22 | .83 |
| FWHM | .07 | .02 | .07 | .01 | .21 | .84 |
| Voxel GM | .63 | .06 | .63 | .07 | .06 | .95 |
| Voxel WM | .33 | .06 | .34 | .07 | -.59 | .56 |
| Voxel CSF | .04 | .03 | .03 | .02 | 1.49 | .15 |
| Glutamate % CRLB | 10.94 | 2.49 | 8.91 | 1.5 | 3.01 | .01 |
| GLx % CRLB | 11.82 | 3.07 | 9.54 | 1.8 | 2.84 | .01 |
| Myo-inositol % CRLB | 6.25 | 1.92 | 5.86 | 1.38 | .74 | .47 |
| NAA % CRLB | 4.59 | 1.94 | 3.97 | .99 | 1.24 | .23 |
| Total choline % CRLB | 5 | 2.74 | 4.29 | 2.52 | .89 | .38 |
| Creatine % CRLB | 4.53 | 1.33 | 3.86 | .65 | 1.98 | .06 |

CHR-good, Clinical High-Risk with a good functional outcome; CHR-poor, Clinical high-risk with a poor functional outcome; signal to noise ratio; FWHM, full-width-half-maximum; GM, grey matter; WM: white matter; CSF, cerebrospinal fluid; CRLB, Cramer-Rao Lower Bounds; Glx, combined measure of glutamine and glutamate; NAA, N-acetylaspartate.

**Supplementary table 5:** Results of the fMRI x ^1^H-MRS glutamate analysis by modality, comparison and lateralisation.

| **Modality** | **Comparison** | | **Significant result** | |
| --- | --- | --- | --- | --- |
| fMRI x ^1^H-MRS glutamate | CHR-good > CHR-poor | | **Right**  *p*FWE =.004, x y z=28 -24 -12, t=4.61, z=4.15, cluster extent=446 | |
|  |  |  | | **Left**  *p*FWE =.008, x y z=-22 -12 -12, t=4.36, z=3.96, cluster extent=468 |

**Supplementary table 6:** Results of the neutral>standard contrast in the fMRI x ^1^H-MRS Glx analysis by modality, comparison and lateralisation.

| **Modality** | **Contrast** |  | **Comparison** | **Significant result** |
| --- | --- | --- | --- | --- |
| fMRI x 1H-MRS Glx | neutral >standard |  | GAF=>65 <  GAF=<65 | **Right**  *p=*.002, x y z =28 -26 -12, t =2.99, z =2.85, cluster extent=17 |
|  |  |  |  | **Left**  *p=*.002, x y z =-24 -24 -14, t =2.98, z =2.83, cluster extent=17 |
